# Association between CD4 Cell Count and Blood Pressure and Its Variation with Body Mass Index Categories in HIV-Infected Patients

**DOI:** 10.1155/2018/1691474

**Published:** 2018-01-22

**Authors:** Christian Akem Dimala, Benjamin Momo Kadia, Ben-Lawrence Kemah, Maxime Tindong, Simeon-Pierre Choukem

**Affiliations:** ^1^Faculty of Epidemiology and Population Health, London School of Hygiene and Tropical Medicine, London, UK; ^2^Department of Orthopaedics, Southend University Hospital, Essex, UK; ^3^Health and Human Development (2HD) Research Network, Douala, Cameroon; ^4^Presbyterian General Hospital Acha-Tugi, Acha-Tugi, Cameroon; ^5^Grace Community Health and Development Association (GRACHADA), Kumba, Cameroon; ^6^Department of Vascular Surgery, Ashford and St Peter's Hospitals NHS, Surrey, UK; ^7^Department of Public Health, Université libre de Bruxelles, Brussels, Belgium; ^8^Diabetes and Endocrinology Unit, Department of Internal Medicine, Douala General Hospital, Douala, Cameroon; ^9^Department of Internal Medicine and Paediatrics, Faculty of Health Sciences, University of Buea, Buea, Cameroon

## Abstract

The aim of this study was to establish whether an independent relationship exists between CD4 count and hypertension and if this relationship is modified or confounded by the body mass index (BMI).* Methods*. A secondary data analysis of a cross-sectional study on 200 HIV/AIDS patients at a referral hospital in Cameroon was conducted. Linear and logistic regression models were used as appropriate to explore the association between the variables of interest.* Results*. There was no linear association between log CD4 count and both systolic (*p* = 0.200; *r* = 0.12) and diastolic blood pressures (*p* = 0.123; *r* = 0.14), respectively. After adjusting for BMI, patients with CD4 count ≥ 350 cells/*μ*l were more likely to have hypertension than those with CD4 count < 350 cells/*μ*l (AOR: 2.50, 95% CI: 1.05–5.93, and *p* = 0.032). There was no effect modification from BMI (test of homogeneity, *p* = 0.721). There was no independent relationship between CD4 count and hypertension after controlling for age, sex, family history of hypertension, BMI-defined overweight, HAART use, and duration of HIV infection (AOR: 1.66, 95% CI: 0.48–5.71, and *p* = 0.419).* Conclusion*. This study did not identify any independent relationship between CD4 count and hypertension. Large prospective studies are recommended to better explore this relationship between hypertension and CD4 count.

## 1. Introduction

The introduction of highly active antiretroviral therapy (HAART) has greatly reduced the morbidity and mortality due to Human Immunodeficiency Virus/Acquired Immunodeficiency Syndrome (HIV/AIDS), with patients experiencing longer and healthier lives [1, 2]. These increases in life expectancy and quality have, however, coincided with an epidemiological transition characterized by an increase in rates of noncommunicable diseases including cardiovascular diseases (CVD) among HIV-infected persons.

Nadir CD4 cell count in HIV-infected patients has been shown to be associated with hypertension [3, 4], through processes of persistent immune activation, chronic inflammation, endothelial dysfunction, and microbial translocation that occurs with inadequate immune recovery [5–9]. The lipid metabolism derangements and accelerated atherosclerosis associated with HAART have also been reported to affect the blood pressure in these patients [10, 11].

The relationship between hypertension and the body mass index (BMI) is well established in immunocompetent subjects with a tendency towards a higher prevalence of hypertension with increasing BMI [12, 13]. The metabolic and immunologic changes associated with the chronic-disease state in HIV-infected patients, not on treatment, predispose them to having suboptimal BMIs. However, there is a significant increase in BMI following initiation of treatment [14–16]. This implies that the association between the CD4 cell count and hypertension could be different across the various BMI categories. This creates an imperative for further exploration of this relationship as it may represent an avenue for intervention in HIV/AIDS patients to reduce adverse outcomes associated with noncommunicable diseases and also to improve the quality of life of these patients.

The aim of this study was therefore to assess if there is an independent relationship between CD4 cell count and hypertension in HIV/AIDS patients and if this association is modified or confounded by the BMI. Specifically we had as objectives to determine if there is a linear association between blood pressure (BP) values and CD4 cell count and to determine if there is an association between hypertension and CD4 cell count categories after controlling for BMI and other important confounders.

## 2. Methods

### 2.1. Study Design and Participants Selection

This was a secondary analysis of data from a cross-sectional study conducted at the Limbe Regional Hospital in Cameroon involving HIV-infected patients receiving care at the HIV treatment center of this health facility [17]. The selection of study participants, study procedures, data sources, and measurements are described in detail in a previous report [17].

### 2.2. Participants and Sampling

Patients coming for regular care and follow-up at this HIV treatment center were recruited randomly (HAART-naïve group) and by consecutive convenient sampling (HAART group matched by age and sex to the HAART-naïve group) to take part in the study. Patients were selected if they were aged 21 and above, had been on HAART for at least 12 months (HAART group), and are with no known previous history of hypertension, diabetes, or renal disease (based on patients' self-reports and medical records). Patients with nonadherence to HAART for more than 6 months and those on medications known to affect blood pressure, corticosteroids, and oral contraceptives were excluded.

### 2.3. Study Procedures and Variables

A face-to-face interview and a physical examination were done for all participants who consented to take part in the study. A structured questionnaire was used for data collection on participants' sociodemographic and clinical characteristics. Data on HIV infection status and duration, previous and most recent CD4 counts less than 3 months old, and lifestyle habits such as smoking, alcohol consumption, and physical activity were collected from the patients and complemented by recorded parameters from their medical records. The physical exam consisted of measuring patient blood pressures, body mass indices, and waist and hip circumferences.

### 2.4. Data Sources and Measurements

Blood pressure was measured on the right arm for all participants while seated using an electronic automated BP monitor (Omron M2, HEM-7121-E) of an appropriate cuff size (22–34 cm). Two measurements were taken at an average interval of 5 minutes and a mean blood pressure was calculated from the two readings. Hypertension was diagnosed according to the World Health Organization (WHO) criteria as systolic BP ≥ 140 mmHg and/or diastolic BP ≥ 90 mmHg [18]. A CD4 cell count below 350 was considered as low based on the WHO recommended threshold for initiation of HAART at the time. CD4 cell count as a continuous variable was thus transformed into a binary variable with categories < 350 cells/*μ*L and ≥ 350 cells/*μ*L. Weight was measured with a scale (BRN 9311) to the nearest half kilogram and patients were allowed to wear just light clothing unlikely to considerably affect the overall weight read. Height was measured with a stadiometer to the nearest half centimeter. Body mass index, calculated as weight (kg)/[height (m) × height (m)], was reported to 2 decimal places in Kg/m^2^. Conventional cut-offs were used to define the various BMI categories as follows: below 18.5 as underweight, 18.5 to 24.9 as normal, 25 to 29.9 as overweight, and above 30 as obesity. Waist and hip circumferences were measured midway between the iliac crest and the lower rib margin and at the intertrochanteric level, respectively. Waist-to-hip ratio, calculated as waist (cm)/hip (cm) circumferences, was reported to the 2 decimal places. Conventional ranges were used to define abdominal obesity derived from the WHR as a WHR > 0.9 in men and WHR > 0.85 in women. Intake of more than three (two for women) standard glasses of wine per day or more than ten (five for women) local beers (1 local beer contains 28 g of alcohol) per week was considered as excessive alcohol consumption. At least 30 minutes of intense physical activity once a week or more was considered as regular physical activity.

### 2.5. Data Management and Data Analysis

STATA version 14.1 statistical software was used to perform the analysis. For objective 1, the CD4 cell count value was log transformed due to its skewed distribution, and a linear regression model was built to assess the association between blood pressure (systolic and diastolic) and log CD4 cell count, both as continuous variables. Linear regression analyses were also done in subgroups of the Log CD4 cell count to identify possible group-specific trends. For objective 2, the Wilcoxon rank sum (Mann–Whitney) test was used to compare the median CD4 cell count in patients with and without hypertension. Also, the Chi-square test was used to assess for any association between hypertension and CD4 cell count as a binary variable across each BMI category, except in situations where Fisher's exact test was most appropriate. For objective 3, a logistic regression model was built to assess the association between hypertension and CD4 cell count while adjusting for BMI, and other confounders. Low *p* values were suggestive of strong evidence of an association between variables, with statistical significance set at a value of *p* < 0.05.

### 2.6. Ethical Considerations

Ethical approval for the primary study was obtained from the Institutional Review Board of the Faculty of Health Sciences of the University of Buea (approval number: 2013/0083/UB/FHS/IRB) and in accordance with the Helsinki Declaration on ethical principles. No ethical clearance was required for the secondary data analysis. All participants found to have elevated blood pressure values were referred to the medical team of the same hospital for further investigation on the possibility of hypertension and management as appropriate. The “Strengthening the Reporting of Observational Studies in Epidemiology” (STROBE) guidelines were used for reporting this study.

## 3. Results

### 3.1. Sociodemographic and Clinical Characteristics of the Study Population

Two hundred eligible participants who consented to participate were recruited. The participants had a mean age of 39.1 ± 9.4 years and 70% were female. The sociodemographic characteristics of the participants are summarized on Table 1. The mean BMI of the participants was 24.1 ± 2.9 Kg/m^2^ with a prevalence of BMI-defined overweight and obesity of 40.5%. The mean WHR was 0.86 ± 0.06 and the median duration of HIV infection in months was 22 months (IQR: 1–68). Fifty percent of the participants had been on first-line antiretroviral therapy for a median duration of 58.6 ± 28.5 months. Fifty-seven participants (28.5%) had hypertension and the median CD4 cell count of this study population was 271 cells/*μ*L (IQR: 130–408). The clinical characteristics of the participants are presented on Table 1.

There was a statistically significant association between BMI as a categorical variable and both hypertension (Fisher's exact, *p* = 0.010) and CD4 cell count (Fisher's exact, *p* = 0.012). The observed trend was an increasing prevalence of hypertension across increasing BMI and a higher BMI with higher CD4 cell count. Factors found to have an association with hypertension were age above 40, male gender, BMI-defined overweight/obesity, HAART use, and HIV infection duration above 30 months (Table 2).

### 3.2. Association between Blood Pressure and CD4 Cell Count

**Figure 1:**
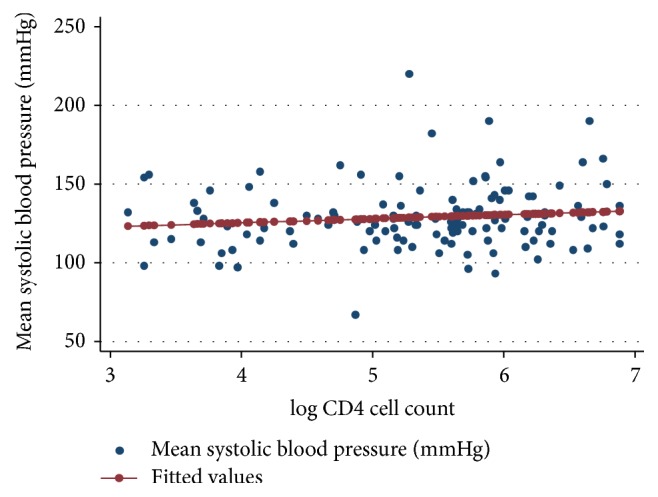
Scatter plot for mean systolic blood pressure and log CD4 cell count.* The red line represents the prediction line of the regression model.*

**Figure 2:**
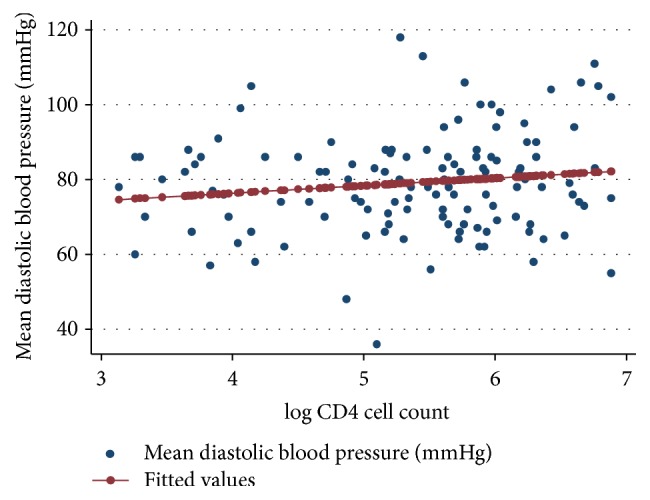
Scatter plot for mean diastolic blood pressure and log CD4 cell count.* The red line represents the prediction line of the regression model.*

Scatter plots for systolic and diastolic blood pressure values against the log CD4 cell count were produced (Figures 1 and 2). From the built linear regression model, there was no linear relationship between the systolic blood pressure (*p* = 0.200, correlation coefficient = 0.12) or the diastolic blood pressure (*p* = 0.123, correlation coefficient = 0.14) and the log CD4 cell count. No significant linear associations were identified between these variables in the subgroups of the log CD4 cell count (Table 3).

### 3.3. Association between Hypertension and CD4 Cell Count across BMI Categories

There was no significant difference in the median CD4 cell count in those with (356 cells/*μ*L (IQR: 182–505)) and those without hypertension (242 cells/*μ*L (IQR: 111–377)), *p* = 0.069. Participants with CD4 cell counts ≥ 350 cells/*μ*L were three times more likely to have hypertension than those with CD4 cell counts < 350 cells/*μ*L (OR: 3.07; 95% CI: 1.32–7.16; *p* = 0.006). There was a reduction in the strength of association between CD4 cell count and hypertension after adjusting for BMI, suggestive of a positive confounding effect by BMI (Mantel Haenszel pooled odds ratio: 2.50) (95% CI: 1.05–5.93, *p* = 0.032) (Table 4). There was no effect modification of this association by BMI (test of homogeneity *p* = 0.721).

On logistic regression adjusted for age, sex, family history of hypertension, BMI-defined overweight, HAART use, and duration of HIV infection, we found no significant association between CD4 cell count and hypertension (AOR: 1.66; 95% CI: 0.48–5.71; *p* = 0.419) (Table 5).

## 4. Discussion

In this study, we aimed to determine if there is an association between blood pressure, hypertension, and CD4 cell count and investigate if this association could vary with changes in BMI. We found no linear association between the log CD4 cell count and blood pressure. A CD4 cell count ≥ 350 cells/*μ*L was associated with higher odds of hypertension, and the strength of this association decreased after adjusting for BMI. This association was completely lost when several other risk factors of hypertension were adjusted for.

There was no linear association between the CD4 cell count and both the systolic and diastolic blood pressure values. Also, there was no statistically significant difference between the median CD4 cell count among participants with and without hypertension. Likewise, the subgroup analysis within intervals of the log CD4 cell count did not identify any linear relationship between these variables. It is worth noting, however, that the nonexistence of an association between the CD4 cell count and blood pressure values from the linear regression model simply means that a change in the CD4 cell count is not associated with a proportional change in the blood pressure, but this does not exclude the possibility of other nonlinear associations between them. This is confirmed by the fact that we later on found an association between the CD4 cell count as a binary variable and hypertension, with CD4 cell counts of 350 cells/*μ*L and above found to be associated with hypertension. The observed association of a higher prevalence of hypertension among patients with high CD4 cell counts is contrary to the findings of the studies by De Socio et al. [3] and Manner et al. [4] in which an independent and significant association was observed between hypertension and nadir CD4 cell count. Nevertheless, the majority of participant in both studies were of a different ethnicity from that of our participants. Even though the prevalence of hypertension in our study was similar to that reported by De Socio et al., half of those hypertension patients in their study were on antihypertensive medications with a third of them being well controlled, and a just small proportion of their participants were on HAART [3]. All these factors could potentially account for the differences in mean blood pressure values and underlying associations between blood pressure and CD4 cell count in these studies and ours.

HIV infection results in a chronic inflammatory, endothelial dysfunction and subsequent impaired elasticity of arteries [19, 20]. HIV infection with and without HAART has been directly associated with aortic stiffness as well [21, 22]. Furthermore, Kaplan et al. reported that a low CD4 cell count is a strong risk factor for carotid atherosclerosis through its association with subclinical carotid artery lesions, even after adjusting for antiretroviral therapy which could be a major confounder of this association [23]. These are all in favor of an association between nadir CD4 cell count and hypertension. Nevertheless, we do note that the possibility of participants in our study having developed hypertension when their CD4 cell counts were low does exist but cannot be ascertained given the study design. On the other hand, Bloomfield et al. [24] in a large retrospective study reported findings similar to ours, as they observed a higher prevalence of hypertension with higher CD4 cell counts among participants aged 35 and below.

Raised BMI, which is associated with hypertension, was found to also have an association with higher CD4 cell counts, making it a potential confounder of the association between hypertension and CD4 cell count. Adjusting for BMI reduced the strength of the association between hypertension and CD4 cell count, indicating a positive confounding effect from BMI. This is understandable given the fact that the risk of having hypertension increases as the BMI increases. A close analysis of the stratum specific odd ratios of the association between hypertension and CD4 cell count across the BMI categories reveals a stronger association among patients with normal BMI compared to overweight patients (Table 4). This finding is, however, as a result of chance given the nonsignificant test of homogeneity of the compared odds ratios.

These observed discrepancies in the study findings suggest hypertension results from a more complex interplay of several environmental and genetic predisposing factors. In this study, older age, male gender, BMI overweight, HAART, and prolonged HIV infection were all found to significantly contribute to having hypertension. As such, any attempt to fully elucidate the direct link between hypertension and CD4 cell count should therefore take into consideration the effect of all these important factors associated with hypertension.

The logistic regression model revealed no association between hypertension and CD4 cell count after adjusting for age, sex, family history of hypertension, BMI-defined obesity, antiretroviral therapy, and duration of HIV infection. This means that the association between hypertension and CD4 cell could exist due to some important confounding.

The interpretation of our results should, however, take into considerations some important limitations of the study. As with all cross-sectional studies, causality cannot be ascertained since, inasmuch as a given CD4 threshold could predispose to hypertension, the presence of hypertension as a comorbidity in HIV-infected patients could predispose them to poor or delayed immune recovery and consequently lower than expected CD4 cell count levels. However, we aimed to study if there existed an association irrespective of its being causal. Nevertheless, prospective cohorts will be better designs to study this association between CD4 cell count and hypertension as serial blood pressure and CD4 cell count measurements over time will enable us to determine at what CD4 cell count values hypertension is most likely to set in. Also the generalizability of these results to settings demographically different from the settings of this study may not be entirely possible. Despite these limitations, this study reiterates the importance of factors associated with hypertension while investigating the effect of HIV infection and CD4 cell count on hypertension.

## 5. Conclusion

This study found no linear association between CD4 cell count and blood pressure. There is no independent relationship between CD4 cell count and hypertension after adjusting for important confounders such as BMI, age, sex, HAART use, and duration of HIV infection. Large prospective studies are therefore recommended to explore this relationship.

## Figures and Tables

**Table 1:** Sociodemographic and clinical characteristics of the participants.

Characteristic	Participants (*n* = 200)
Age in years (mean ± SD)	39.1 ± 9.4
Females, *n* (%)	140 (70%)
Married, *n* (%)	89 (44.5%)
Unskilled occupation, *n* (%)	26 (13%)
Northwest region, *n* (%)	88 (44%)
BMI in Kg/m^2^ (mean ± SD)	24.1 ± 2.9
BMI-defined overweight and obesity (prevalence, %)	40.5%
BMI categories	
Underweight	2 (1.0%)
Normal	117 (58.5%)
Overweight	81 (40.5%)
WHR (mean ± SD)	0.86 ± 0.06
WHR-defined abdominal obesity (prevalence, %)	44.5%
Hypertension in mmHg (prevalence, % (95%CI))	28.5% (22.4–35.3)
Systolic blood pressure (mean ± SD)	128 ± 20
Diastolic blood pressure (mean ± SD)	79 ± 13
Duration of HIV infection in months, median (IQR)	22 (1–68)
CD4 cell count in cells/*μ*L, median (IQR)	271 (130–408)
WHO clinical stage, *n* (%)	
Stage I	23 (11.5%)
Stage II	62 (31.0%)
Stage III	98 (49.0%)
Stage IV	17 (8.5%)

**Table 2:** Factors associated with hypertension in the study population.

Factor	Participants (*n* = 200)	Participants with HTN (*n* = 57)	*p* value
Age (in years)			
>40	78	35 (44.9%)	**<0.001**
≤40	122	22 (18.0%)	
Gender			
Male	60	26 (44.3%)	**0.002**
Female	140	31 (22.1%)	
Family history of HTN			
Yes	11	1 (9.1%)	0.142
No	189	56 (29.6%)	
Smoking			
Yes	11	6 (54.6%)	**0.049**
No	189	51 (27.0%)	
Alcohol consumption			
Yes	52	14 (26.9%)	0.767
No	148	43 (29.1%)	
Physical exercise			
Yes	42	12 (28.6%)	0.990
No	158	45 (28.5%)	
BMI-defined overweight/obesity			
Yes	81	32 (39.5%)	**0.004**
No	119	25 (21.0%)	
WHR-defined overweight/obesity			
Yes	89	24 (27.0%)	0.667
No	111	33 (29.7%)	
HAART use			
Yes	100	38 (38.0%)	**0.003**
No	100	19 (19.0%)	
Duration of HIV infection (months)			
>30	92	35 (38.0%)	**0.006**
≤30	108	22 (20.4%)	

BMI: body mass Index, HTN: hypertension, and WHR: waist-to-hip ratio.

**Table 3:** Linear regression model for systolic and diastolic blood pressures and log CD4 cell count.

Parameter	Regression coefficient	Intercept	*p* value	*R*-square	Correlation coefficient
Overall (*n* = 123)					
Systolic BP	2.55	115.17	0.200	0.0136	0.1165
Diastolic BP	2.01	63.30	0.123	0.0196	0.1398
Log CD4: >3-4 (*n* = 16)					
Systolic BP	−28.70	225.84	0.121	0.1632	−0.4039
Diastolic BP	−0.24	78.20	0.981	0.0	−0.0066
Log CD4: >4-5 (*n* = 19)					
Systolic BP	−11.73	180.13	0.466	0.0317	−0.1779
Diastolic BP	−4.62	97.64	0.664	0.0114	−0.1067
Log CD4: >5-6 (*n* = 56)					
Systolic BP	4.23	106.57	0.682	0.0031	0.0559
Diastolic BP	5.47	48.10	0.400	0.0132	0.1147
Log CD4: >6-7 (*n* = 32)					
Systolic BP	8.47	76.62	0.504	0.0150	0.1227
Diastolic BP	5.75	45.51	0.555	0.0117	0.1084

**Table 4:** Association between CD4 and hypertension across BMI categories, *n* = 123.

BMI groups and CD4 categories	Hypertension	Odds ratios (95% CI)	*p* value^*∗*^
All participants			
<350	15/78 (19%)	1	**0.006**
≥350	19/45 (42%)	3.07(1.32–7.16)^*∗∗*^	
Normal BMI			
<350	8/55 (15%)	1	0.066
≥350	7/21 (33%)	2.94 (0.87–9.87)	
Overweight			
<350	7/22 (32%)	1	0.211
≥350	12/24 (50%)	2.14 (0.62–7.38)	
MH controlled for BMI	-	2.50(1.05–5.93)^*∗∗∗*^	0.032

^*∗*^Chi-square test.  ^*∗∗*^Crude odds ratio.  ^*∗∗∗*^Mantel Haenszel pooled odds ratios, the test of homogeneity of odds ratios, and *p* = 0.721.

**Table 5:** Association between CD4 cell count and hypertension after controlling for confounders.

Factor and categories	Adjusted odds ratio^*∗*^	95% confidence interval	*p* value^*∗∗*^

CD4 cell count (≥350/<350)	1.66	0.48–5.71	0.419
Age (>40/≤40 years)	1.76	0.70–4.39	0.229
Gender (Male/Female)	2.77	1.09–7.03	**0.032**
Family History of HTN (Yes/No)	0.34	0.03–3.69	0.373
BMI-defined obesity (Yes/No)	2.75	1.09–6.91	**0.031**
HAART (Yes/No)	1.32	0.21–8.29	0.765
HIV duration (>30/≤30)	1.30	0.21–7.96	0.773

^*∗*^Odds ratio adjusting for all variables on the table;  ^*∗∗*^*p* values for the Wald test statistic. BMI: body mass index, HAART: highly active antiretroviral therapy, and HTN: hypertension.
